# *EML4-ALK*融合基因阳性肺腺癌合并淋巴瘤1例并文献复习

**DOI:** 10.3779/j.issn.1009-3419.2015.02.07

**Published:** 2015-02-20

**Authors:** 丽 刘, 伟 衡

**Affiliations:** 215006 苏州，苏州大学附属第一医院呼吸内科 Department of Respiratory Medicine, The First Affiliated Hospital of Soochow University, Suzhou 215006, China

**Keywords:** 肺肿瘤, 淋巴瘤, 治疗, 第二原发癌, 间变性淋巴瘤激酶, Lung neoplasms, Lymphoma, Treatment, Second primary carcinoma, Anaplastic lymphoma kinase

## Abstract

近年来随着分子生物学研究的不断深入，靶向治疗成为当前肺癌治疗的趋势。目前肺癌个体化的最佳治疗效果日益受到重视，棘皮动物微管相关蛋白4-间变型淋巴瘤激酶（echinoderm microtubule associated protein like 4 anaplastic lymphoma kinase, *EML4-ALK*）融合基因作为新兴生物标记物是当前肺癌治疗领域研究热点。与此同时，随着抗肿瘤治疗水平的不断提高，生存期明显延长，发生多原发癌（multiple primary carcinomas, MPC）的机会增多。*EML4-ALK*融合基因阳性的肺腺癌合并淋巴瘤发生于同一患者文献报道罕见。本文报道1例*ALK*融合基因阳性的非小细胞肺癌（non-small cell lung cancer, NSCLC）合并淋巴瘤病例，同时对异时性肺癌合并淋巴瘤的文献进行复习。

## Abstract (en)

In recent years, with the deepening of the research of molecular biology, targeted therapy has become one of the trend of lung cancer treatment. The individualized treatment of lung cancer is attached great importance at present. Echinoderm microtubule associated protein like 4 anaplastic lymphoma kinase (EML4-ALK) as a new biological marker is a hot topic in the field of lung cancer treatment. Meanwhile, with the improvement of anticancer treatment and survival, the incidence of multiple primary carcinomas (MPC) has become increasingly. But the report that malignant lymphoma complicated with lung adenocarcinoma harboring *EML4-ALK* fusion gene in one individual is rare. Here, we report an EML4-ALK positive non-small cell lung cancer (NSCLC) in a patient previously diagnosed with T cell lymphoma and review literature on metachronous lung cancer complicating with lymphoma.

淋巴瘤是最早发现的血液系统恶性肿瘤之一，随着诊治水平的不断提高，患者生存期的相对延长，淋巴瘤患者发生第二原发癌（second primary carcinoma, SPC）的发病风险在增加，SPC以血液系统肿瘤、胃肠道、乳腺多见，而肺癌的发生率约为1/100。本文报道1例间变型淋巴瘤激酶（anaplastic lymphoma kinase, *ALK*）融合基因阳性的肺腺癌合并淋巴瘤的病例。

## 1 临床资料

2012年5月，48岁的男性患者出现咳嗽，伴乏力、活动后气喘，未予重视。9月至中医院就诊，全胸片示“肺部感染”，抗感染治疗后复查胸片：肺部炎症吸收。此后约半年患者间断咳嗽伴乏力，活动后气喘，2013年4月就诊我科门诊，胸部计算机断层扫描（computed tomography, CT）示：右肺门处占位，肺门及纵隔淋巴结肿大，心包积液。支气管镜检查病理示：（支气管粘膜）癌，类型倾向粘液表皮样癌。患者于2013年4月24日收入院。1984年经颈部淋巴结活检及骨穿诊断：T细胞源性淋巴瘤，曾行CHOP方案（环磷酰胺+长春新碱+阿霉素+强的松）、COMP方案（环磷酰胺+长春新碱+甲氨蝶呤+强的松）和COMP+CCNU方案（环磷酰胺+长春新碱+甲氨蝶呤+强的松+卡莫司汀）化疗，并接受放疗，经腰穿行甲氨蝶呤鞘内注射化疗，已治愈。吸烟史30年，1包/d，已戒烟1年。入院相关检查：肿瘤全套、腹部B超、头颅磁共振成像（magnetic resonance imaging, MRI）未见异常。骨扫描：右侧肩关节、T10及右侧骶髂关节反应性骨形成活跃。诊断：右肺腺癌Ⅳ期（T4N2M1）。表皮生长因子受体（epidermal growth factor receptor, *EGFR*）基因检测结果示：18、19、20、21外显子无突变。患者于2013年5月起，先后接受5个疗程培美曲塞联合顺铂化疗，期间多次评估病情：部分缓解（partial response, PR），患者存在间断发热咳嗽喘息及消化道不良反应，予对症及支持治疗。后行4个疗程的培美曲塞500 mg/m^2^单药维持治疗，期间评估病情：病情稳定。2014年1月患者出现骨痛，经同位素骨扫描：全身多发转移性骨损害，予唑来膦酸及对症治疗。2014年3月1日复查胸部计算机断层扫描（computed tomography, CT）检查，显示肺部病灶进展，患者咳嗽、痰血、气急明显，再次经气管镜检查获取组织，荧光原位杂交（florescence *in situ* hybridization, FISH）方法检测棘皮动物微管相关蛋白4-ALK（echinoderm microtubule associated protein like 4 ALK, *EML4-ALK*）融合基因：EML4-ALK变异体1、2、3：阳性，予克唑替尼250 mg *bid*治疗，治疗3 d后咳嗽胸闷减轻，痰血消失，期间主要不良反应轻度肝损和间断腹泻、纳差乏力，无明显视觉异常，经对症处理后好转。患者服药40 d后CT显示病灶显著吸收，按实体瘤疗效评价标准（Response Evaluation Criteria in Solid Tumors, RESIST）标准获得PR，此后6个月和9个月分别复查胸部CT，病灶进一步较前缩小，病情持续改善。患者目前仍定期我科随访中，在诊断肺癌17个月后重新走上工作岗位。

## 2 讨论

早在1869年Billroth首次提出多原发癌（multiple primary carcinomas, MPC），直到1932年Warren和Gates提出了MPC的诊断标准，关于MPC逐渐被认识到。MPC系重复癌，是指2种或2种以上不同性质的原发性恶性肿瘤同时或先后发生于同一个体^[1]^。关于MPC发病率国内外报道不同，国外报道MPC发生率为1.6%-10.7%，国内报道为0.35%-2.4%^[2]^。Konits等^[3]^认为，肺癌是淋巴瘤患者延长生存的主要并发症之一。据报道，淋巴瘤患者发生肺癌的风险是普通人群的3倍多。关于淋巴瘤患者发生SPC的病因目前还不明确。可能与淋巴瘤患者免疫功能低下，使癌细胞以较快的速度生长有关。大多数文献认为淋巴瘤的治疗会诱发SPC，可能是抗肿瘤治疗的同时也存在致癌作用。Van Leeuwen等^[4]^统计了18年间744例霍奇金淋巴瘤（Hodgkin' s lymphoma, HL）患者后续发生14例肺癌，其发生肺癌的风险与大剂量放疗有关，尽管所有的肺癌都发生在非放射部位。Travis等^[5]^对29, 153例非霍奇金淋巴瘤（Non-Hodgkin' s lymphoma, NHL）患者进行回顾性分析，亦发现放疗增加了肺癌的发生风险。在随访655例HL或NHL患者中发现6例肺癌，指出淋巴瘤的放化疗联合治疗可能增加了肺癌的发生风险，单纯化疗增加肺癌的发生风险，其所起的作用是较小的，而与放疗结合后更明显^[3]^。化疗药物如长春新碱、阿霉素等的应用容易诱发已接受放疗的肺组织病变。烷化剂的使用致使SPC的发生增加。本报道中，患者治疗淋巴瘤时应用CHOP方案后，应用卡莫司汀联合COMP方案治疗淋巴瘤复发，与文献报道的相关化疗药物一致，同时亦接受放疗，可能是放化疗的结合增加了SPC的发病风险。从发生的时间上，MPC可分为同时性和异时性，以后者居多。间隔时间的报道国内外不尽相同，最长的可达43年，且MPC间隔时间越长，预后越好。Lorenzo Bermejo等^[6]^对60, 901例NHL患者进行分析，SPC的相对风险是随时间变化的U型曲线，且相对风险最高时发生于诊断第一原发癌20年后。SPC的发病因素亦可能与遗传、理化因素、不良生活习惯、机体易感、种族、基因突变等有关。有关吸烟与淋巴瘤合并SPC为肺癌的相关性，目前文献报道不一致，有待于进一步研究。

目前肺癌的个体化治疗日益受到重视，除EGFR外，*EML4-ALK*融合基因为2007年发现的肿瘤相关基因^[7]^，明确为NSCLC的驱动基因之一。ALK最早发现于间变性大细胞淋巴瘤（anaplastic large cell lymphoma, ALCL），是由于2号染色体上的*ALK*基因位点的畸变，形成融合基因*NPM-ALK*。其特征鲜明，主要发生在30岁之前，性别差异明显，男女比率达6:1，ALK阳性（ALK+）的ALCL预后明显好于ALK阴性（ALK-）的病例，ALK+ ALCL较好的预后可能与肿瘤增殖率高、化放疗敏感有关，治愈的可能性最大。本例患者30年前罹患T细胞淋巴瘤接受放化疗治疗后，达到临床治愈，符合ALK，淋巴瘤特点。鉴于30年前的诊断水平，故ALK表达情况未知。现有资料^[8]^表明*EML4-ALK*融合在非小细胞肺癌（non-small cell lung cancer, NSCLC）中的发生率约为3%-5%。主要发生于较年轻、轻度吸烟/不吸烟、粘液性腺癌，化疗效果不佳者。目前检测*EML4-ALK*融合基因的技术，主要包括FISH、逆转录聚合酶链反应（reverse transcription polymerase chain reaction, RT-PCR）、免疫组织化学技术（immunohistochemistry, IHC）。本例患者接受克唑替尼治疗后，病灶较前显著缩小，表明克唑替尼治疗晚期ALK阳性NSCLC的疗效可靠。Profile 1014临床研究结果表明，克唑替尼一线治疗ALK阳性晚期NSCLC的无进展生存期（progression-free survival, PFS）为10.9个月，而化疗组的PFS为7.0个月，靶向治疗组获益显著，本例患者接受含铂两药诱导化疗及单药维持治疗共9个周期后，疾病进展，二线服用克唑替尼，病情依然快速获得缓解，与Profile 1007报道的二线治疗研究结果相符^[9]^。因此，重视基因检测应贯穿于晚期NSCLC治疗的始终，不要轻易放弃靶向治疗的可能，无论一线还是二线治疗，克唑替尼用于ALK阳性NSCLC均疗效确切，不良反应可控。目前关于克唑替尼出现耐药的病例已有报道，一例ALK阳性肺腺癌患者在应用克唑替尼5个月后出现耐药，对其进行研究可能与点突变如C1156Y、L1196M影响基因融合有关，亦可能与激活旁路信号传导、*ALK*基因拷贝数增加有关^[10]^。耐药后持续给予克唑替尼仍然具有临床获益，因ALK信号通路可持续被有效抑制。2013年世界肺癌大会（World Conference on Lung Cancer, WCLC）报道了Ⅰ期和Ⅱ期临床试验，120例患者疾病进展后，继续使用克唑替尼治疗与停药的患者相比，继续使用克唑替尼组的客观缓解率（objective response rate, ORR）和总生存期（overall survival, OS）显著提高^[11]^。研究显示病情再次进展后使用二代ALK抑制剂仍可获得6.9个月的PFS。

ALK阳性肺腺癌和淋巴瘤的基因变异有无关联尚不得而知，异时性多原发癌的病因值得进一步探究，对临床而言，NSCLC的个体化治疗必须以贯穿始终的基因检测为基础，才能使患者最大化生存获益。
